# Supplementary material for: The Hydrolytic Peptides of Soybean Protein Induce Cell Cycle Arrest and Apoptosis on Human Oral Cancer Cell Line HSC-3
Source: Molecules. 2022 Apr 29;27(9):2839. doi: 10.3390/molecules27092839 (PMC9101267; doi:10.3390/molecules27092839)
Supplement: Supplementary file 1 [file molecules-27-02839-s001.zip › molecules-1671119-supplementary.pdf]

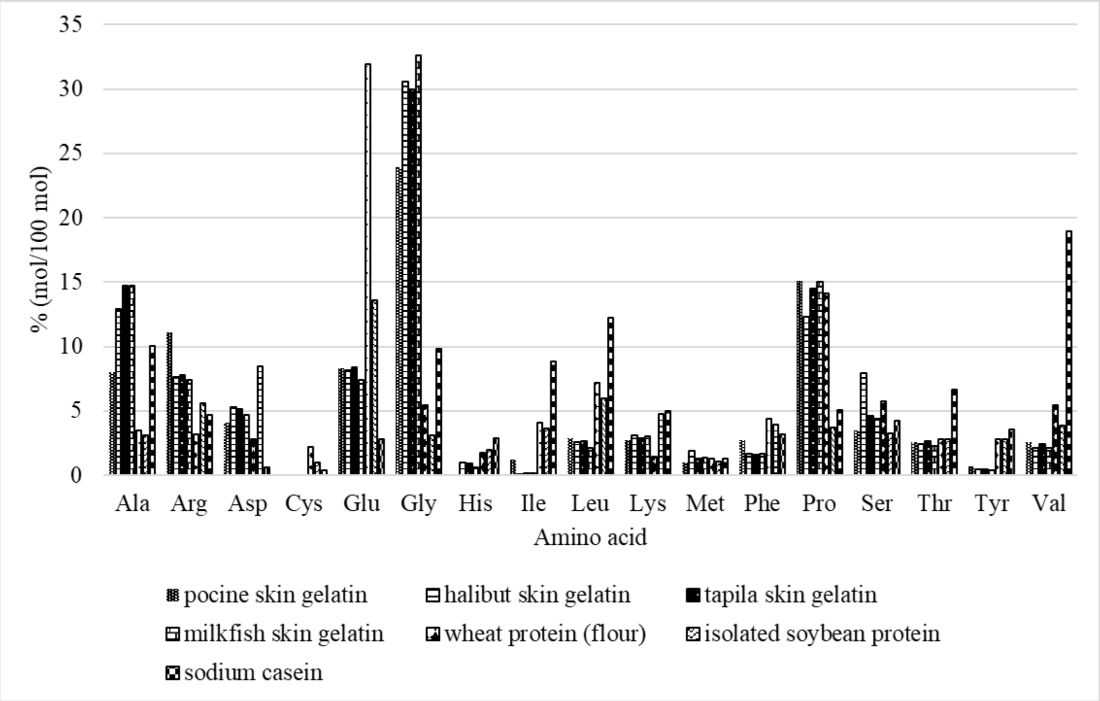

Figure S1. Comparison of average whole amino acid composition of porcine skin gelatin, halibut skin gelatin, tilapia fish skin, milkfish skin gelatin, wheat gluten, soy protein isolate and sodium caseinate. Source of amino acid composition data was cited from Hafidz et al. (2011) (porcine skin gelatin), Wang et al. (2015) (fish skin gelatin), Rombouts et al. (2009) (wheat gluten), Cervantes-Pahm and Stein (2010) (soy protein isolate), Mackle et al. (1999) (sodium caseinate).
